# Condition of bob veal calves on arrival at an abattoir in Ohio

**DOI:** 10.1017/awf.2022.8

**Published:** 2023-01-27

**Authors:** Zachary A England, Hannah L Maggard, Andrew D Fisher, Natalie W Roadknight, Jessica A Pempek

**Affiliations:** 1 Department of Animal Sciences, The Ohio State University, Columbus, OH 43210, USA; 2 Animal Welfare Science Centre, The University of Melbourne, Corner Flemington Road and Park Drive, Parkville, VIC 3052, Australia; 3 Faculty of Veterinary and Agricultural Sciences, The University of Melbourne, 250 Princes Highway, Werribee, VIC 3030, Australia

**Keywords:** animal welfare, bob veal calf, failed transfer of passive immunity, harvest, health, hypoglycaemia

## Abstract

Previous research has shown surplus dairy calves arrive at ‘formula-fed’ veal operations in North America in sub-optimal condition; however, little is known about the condition of ‘bob’ veal calves on arrival at abattoirs. The objectives of this study were to assess the condition of bob veal calves on arrival at an abattoir in Ohio and determine risk factors for poor health outcomes. On arrival, 35 calves in each of 12 cohorts (n = 420 calves) were assessed using a standardised health examination. A blood sample was also collected to assess failed transfer of passive immunity (FTPI) and hypoglycaemia. Descriptive statistics were used to describe the prevalence of poor health outcomes. Mixed-effects logistic regression models were used to identify if calf breed, sex, or source were risk factors for poor health outcomes. The most common physical health concern observed on arrival at the abattoir was dehydration (mean: 68.6%), followed by thin body condition (39.8%), and navel inflammation (25.7%). Approximately one-quarter (23.4%) of calves had FTPI and 73.4% were hypoglycaemic. Male calves were more likely than females to arrive hypoglycaemic. Hydration status was associated with breed; Jersey and crossbreed calves were less likely to be dehydrated than Holstein-Friesian calves. Buying station tended to be associated with FTPI. These results underline the need for more studies investigating morbidity, mortality, and their underlying risk factors to promote calf welfare prior to slaughter in each stage of the production chain: on the dairy farm of birth, during marketing, and in transit.

## Introduction

The United States dairy industry is composed of nearly 9.4 million dairy cows (USDA – National Agricultural Statistics Service 2022), resulting in approximately 9.4 million dairy calves born annually. While one-third of dairy calves typically remain on the farm of birth as replacements for the lactating herd, an estimated 6 million calves are sold annually as ‘surplus’ to the requirements of dairy production (Edwards-Callaway *et al.* 2019; Bolton & von Keyserlingk 2021). Surplus dairy calves are generally sold from the dairy farm of birth within the first week of life (Shivley *et al.* 2019) and destined for ‘bob’ veal (harvested < 3 weeks of age), ‘formula-fed’ veal (harvested at approximately 20 weeks of age; USDA FSIS 2013), or dairy beef (harvested at 12 to 14 months of age; Fanatico 2010).

Notwithstanding their destination, surplus dairy calves enter a rather disaggregated production system, beginning with calf management at the dairy farm of birth and ending at an abattoir (e.g. bob veal) or calf-rearing facility (e.g. formula-fed veal or dairy beef), with many calves experiencing long-distance transportation and co-mingling with other calves or species at live auction or buying stations (i.e. facilities where large groups of calves are aggregated) in between (for a review, see Creutzinger *et al.* 2021). Previous research has documented that formula-fed veal calves arrive at rearing facilities in North America in poor health, with clinical signs of dehydration, diarrhoea, depression, navel inflammation, and failed transfer of passive immunity (FTPI) due to sub-optimal colostrum management practices on the dairy farm of birth (Canada: Renaud *et al.* 2018a; United States: Pempek *et al.* 2017). Recently, Roadknight and colleagues (2021a) used blood parameters to assess the welfare status of bob veal calves on arrival at commercial abattoirs in Australia and reported a majority of calves had blood glucose concentrations similar to calves fasted for 14 to 25 h. Little to no research, however, has assessed the condition of bob veal calves on arrival at abattoirs in the United States. With differences in calf transportation regulations and management practices between countries, it is important to investigate this sector of surplus calf production in a US context.

The objectives of this study were to: (i) assess the condition of surplus dairy calves destined for bob veal on arrival at an abattoir in Ohio; and (ii) determine risk factors for poor health outcomes on arrival.

## Materials and methods

### Study animals, handling and facilities

This cross-sectional cohort study was conducted at an abattoir in Northeastern Ohio, in accordance with the guidelines set forth by The Ohio State University’s Institutional Animal Care and Use Committee (Animal Use Protocol 2021A00000047). This abattoir was selected based on proximity to The Ohio State University, willingness to participate in the study, and because they regularly processed a large number of bob veal calves. A sample size of 420 (35 calves per cohort) was selected, assuming a 50% prevalence and based on data from an earlier study that estimated the prevalence of poor health outcomes in formula-fed veal calves on arrival at calf-rearing facilities (Pempek *et al.* 2017) to estimate the prevalence at a 95% confidence level with an error of less than 5%.

Data collection took place between June and September 2021. Blood samples and clinical health data from 420 calves across 12 cohorts were collected within 2 h after calves arrived at the abattoir (Table 1); a cohort was defined as a group of calves available for evaluation at a specific time-point (i.e. those calves that arrived at the abattoir during the morning of each experimental day) (Hudson *et al.* 2005). The total number of calves in each cohort arriving at the abattoir varied (mean [± SD] of 172.5 [± 54.7]), and 35 calves were sampled from each cohort. Systematic random sampling was utilised, whereby the total number of calves per cohort was considered at arrival, and every *nth* calf was enrolled in the study to ensure an accurate representation of the cohort. Study personnel (two animal welfare scientists, one veterinary student, and one graduate student) with extensive experience handling and assessing calf health performed all aspects of data collection.

**Table 1.** Cohort-level information for bob veal calves arriving at an abattoir in Ohio; Buying Station 1 was located in New York (431 miles from abattoir); Buying Station 2 was located in Pennsylvania (255 miles); and Buying Station 3 was located in Ohio (7 miles)

Cohort	Month	Calves arriving (n)^1^	Dead on arrival (n)	Euthanased (n)	Source
1	June	150	0	2	Buying Station 1
2	June	108	3	0	Buying Station 1
3	July	71	0	0	Buying Station 2
4	July	154	2	1	Buying Station 2 Buying Station 3
5	July	178	0	1	Buying Station 2 Buying Station 3
6	July	237	1	4	Buying Station 2
7	July	64	0	0	Buying Station 2
8	August	127	0	0	Buying Station 2
9	August	176	0	2	Buying Station 2 Buying Station 3
10	August	215	0	3	Buying Station 2 Buying Station 3
11	August	202	1	6	Buying Station 2 Buying Station 3
12	September	206	2	1	Buying Station 2 Buying Station 3

1 Thirty-five calves were randomly sampled from each cohort.

### Clinical health examination

Two members of the research team (ZE and MM) completed all clinical health examinations, using a standardised health scoring system adapted from previous research with young calves. Prior to the onset of the study, ZE and MM assessed the health of 36 calves to ensure consistency between observers for all health outcomes (inter-rater reliability: 92.8%). Health examinations included evaluation for fever (≥ 39.4°C), signs of respiratory disease (four-point scale for eyes and nose discharge and ear droop; McGuirk & Peek 2014), broken ribs or tail (two-point scale), arthritis (four-point scale; Garcia *et al.* 2022), diarrhoea (two-point scale; adapted from McGuirk 2008), navel inflammation (four-point scale; Pempek *et al.* 2017), depression (four-point scale; Pempek *et al.* 2019), body condition (five-point scale; Renaud *et al.* 2018a), and dehydration (skin tent test, four-point scale; Garcia *et al.* 2022). Scoring systems for each health outcome are further described in Table 2.

**Table 2.** Description of scoring criteria used to evaluate calves for signs of respiratory infection (as indicated by eyes and nose discharge and ear droop), broken body parts, joint inflammation, diarrhoea, navel inflammation, dehydration, depression, and poor body condition on arrival at an abattoir in Ohio

	Score				
Variable	0	1	2	3	4
Eye discharge	Normal	Small amount of unilateral ocular discharge	Moderate amount of bilateral ocular discharge	Heavy bilateral ocular discharge	–
Ear droop	Normal	Ear flick or head shake	Slight unilateral droop	Head tilt or bilateral droop	–
Nose discharge	Normal serous discharge	Small amount of unilateral cloudy discharge	Bilateral, cloudy or excessive mucus discharge	Copious bilateral mucopurulent discharge	–
Broken ribs/tail	No broken ribs or tail	Presence of broken rib or tail	–	–	–
Arthritis	No swelling; Not warm or painful	Slight swelling; Not warm or painful	Swelling with pain or heat; Slight lameness	Swelling with severe pain, heat and lameness	–
Diarrhoea	Normal, semi-formed, pasty, or loose but stays on top of bedding	Watery, sifts through bedding	–	–	–
Navel inflammation	Normal (pencil size); No heat, swelling, or discharge	Bigger than normal (width of the pointer finger); No heat, swelling, or discharge	Bigger than normal (width of the pointer and middle fingers combined); Slight pain or moisture	Bigger than normal (width of the pointer, middle, and ring fingers combined); Heat, pain, or malodorous discharge	–
Dehydration	Normal; Eyes are bright, and skin feels pliable	Mild dehydration; Slight loss of skin elasticity; Skin tent <3 s; Eyes not recessed into orbit	Moderate dehydration; Skin tent >3 s but <10 s; Eyes slightly recessed into orbit	Severe dehydration; Skin tent >10 s; Eyes markedly recessed into orbit	–
Depression	Normal; No signs of depression	Mild depression; Calf suckles but not vigorously	Moderate depression; Calf is able to stand, but suckling is weak or disorganised	Severe depression; Calf unable to stand or suckle	–
Body condition	Emaciated with little muscle or fat present and clearly defined bone structure	No subcutaneous fat covering frame	Bony prominences are easily palpated	Thin covering of subcutaneous fat over body prominences	Subcutaneous fat covering bony prominences

### Blood collection, handling and processing

For each calf, a blood sample from the jugular vein was collected into 10-mL vacuum tubes without anticoagulant (BD Vacutainer® Red Top Blood Collection Tubes; Becton Dickinson, Franklin Lakes, NJ, USA).

#### Blood glucose measurements

Immediately following blood collection, blood glucose concentrations were analysed calf-side at the abattoir using a human-based point-of-care blood glucose meter (Contour Next One Meter; Ascensia Diabetes Care, Parsippany, NJ, USA) with single-reagent test-strips (Contour Next One, Ascensia Diabetes Care); this glucose meter was previously validated for measuring calf blood glucose using whole blood (threshold, 4.95 mmol L^–1^; sensitivity, 95.6%; specificity, 80.3%; Renaud *et al.* 2022).

#### Serum total protein measurements

After completion of the calf-side blood glucose measurements, blood samples were placed into a cooler with ice packs, where they remained during transport to The Ohio State University for serum total protein (STP) measurements. Serum was separated by centrifuging the blood samples at 1,200 g for 10 min. One drop of serum was then transferred onto a DD-2 Digital-Dairy Refractometer (MISCO, Solon, OH, USA) to evaluate STP. Serum total protein concentrations were categorised as: excellent (> 6.2 g dL^–1^), good (6.1 to 5.8 g dL^–1^), fair (5.7 to 5.1 g dL^–1^), and poor or FTPI (< 5.1 g dL^–1^; Lombard *et al.* 2020).

### Risk factors for poor condition on arrival

Calf sex was assessed visually and recorded for each calf. Calf breed was also assessed based on phenotype as either Holstein-Friesian, Jersey, crossbreed, or ‘other’ breed. Transport distance was estimated from the live auction or buying station to the abattoir; however, the exact route and time of for each cohort is unknown, as this information was not recorded by the abattoir. The proportion of calves that were dead or euthanased at arrival by abattoir personnel were recorded separately at the cohort-level.

Clinical health assessment scores were dichotomised for analysis. Health assessment scores were considered clinically ‘normal’ if broken ribs or tail score = 0; dehydration score = 0; depression score = 0; faecal score = 0; Body Condition Score ≥ 2; STP ≥ 5.1 g dL^–1^; rectal temperature < 39.4°C; blood glucose ≥ 4.95 mmol L^–1^; joint score = 0 or 1; navel score = 0 or 1; and eyes, ears, or nose score = 0 or 1. Calves were considered to have ‘poor health’ outcomes if broken ribs or tail score = 1; dehydration score > 1; depression score > 1; faecal score = 1; Body Condition Score = 0; STP < 5.1 g dL^–1^; rectal temperature ≥ 39.4°C; blood glucose < 4.95 mmol L^–1^; joint score = 2 or 3; navel score = 2 or 3; and eyes, ears, or nose score = 2 or 3. Table 3 summarises the clinically relevant cut-off values for each variable.

**Table 3.** Clinically relevant cut-points for health parameters assessed in calves on arrival at an abattoir in Ohio

Variable	Health outcome	Normal health outcome	Poor health outcome
Rectal temperature	Fever	< 39.4°C	≥ 39.4°C
Eye discharge	Respiratory disease	0 or 1	2 or 3
Ear droop	Respiratory disease	0 or 1	2 or 3
Nose discharge	Respiratory disease	0 or 1	2 or 3
Broken ribs or tail	Broken ribs or tail	0	1
Arthritis	Arthritis	0 or 1	2 or 3
Diarrhoea	Diarrhoea	0	1
Navel inflammation	Navel inflammation	0 or 1	2 or 3
Dehydration	Dehydration	0	1, 2, or 3
Depression	Depression	0	1, 2, or 3
Body condition	Thin body condition	1 2, 3, or 4	0
Serum total protein concentration	Failed transfer of passive immunity	≥ 5.1 g dL^–1^	< 5.1 g dL^–1^
Blood glucose concentration	Hypoglycaemia	≥ 4.95 mmol L^–1^	< 4.95 mmol L^–1^

To estimate the prevalence of poor health outcomes on arrival at the abattoir, descriptive statistics were generated for all variables in the dataset. Blood glucose samples were not obtained for two cohorts, and one blood sample from another cohort was missing; therefore, this portion of the analysis consisted of 349 observations and ten cohorts. Confidence intervals (CI) for prevalence estimates were calculated using the SURVEYFREQ procedure of SAS (Version 9.4; SAS Institute Inc, Cary, NC, USA), with cohort specified as the cluster variable to account for the expected clustering of observations within cohorts. The EXACT statement was specified when prevalence estimates were < 5%.

To determine if breed, sex, or source were risk factors for poor health outcomes on arrival at the abattoir, mixed-effects logistic regression models (PROC GLIMMIX; SAS, Version 9.4) were used. The hierarchical model included multilevel data, whereby calf (e.g. breed, sex) and cohort level (e.g. source) data were considered. A random intercept was specified for cohort to account for the expected clustering of observations within cohorts, and the hierarchical structure was specified using cohort as the subject. Variables (e.g. breed, sex, source) were offered to the model and retained if the univariable *P*-value was < 0.20. Significant differences were declared at *P* ≤ 0.05 and a trend at 0.05 > *P* ≤ 0.10.

## Results

### Prevalence of poor health outcomes on arrival

On arrival at the abattoir, nearly all (95.5%; 401/420) calves had at least one poor health outcome, and 82.1% (345/420) had two or more poor health outcomes. Across all cohorts (n = 2,070 calves total), 20 calves (mean of 1.7 [± 1.9] per cohort) were dead on arrival at the abattoir, and nine (mean of 0.75 [± 1.1]) were euthanased following arrival by abattoir personnel (Table 1).

The majority of calves were hypoglycaemic (73.9%, 95% CI: 66.6 to 81.3%) on arrival at the abattoir, using a cut-off of 4.95 mmol L^–1^. In addition, 68.6% (95% CI: 57.0 to 80.1%) were considered dehydrated using a skin tent test, 39.8% (95% CI: 26.1 to 53.1%) had thin body condition, and approximately one out of every four calves (25.7%, 95% CI: 21.8 to 29.6%) had navel inflammation. Overall, 17.1% (95% CI: 12.4 to 21.9%) were depressed; however, 0 calves were moribund or unable to rise. Nearly one-quarter (23.4%, 95% CI: 18.5 to 28.3%) of calves had poor transfer of passive immunity (TPI) or FTPI, using a cut-off of 5.1 g dL^–1^. Further, according to recent consensus recommendations for calf-level transfer of passive immunity (Lombard *et al.* 2020), 29.4% (123/419) of calves had fair TPI, 17.9% (75/419) had good TPI, and 29.4% (123/419) had excellent TPI. The number and percentage of calves with normal and poor health outcomes are presented in Table 4.

**Table 4.** Number and percentage of calves with clinically normal and poor health outcomes (95% CI) from a sample of 420 bob veal calves on arrival at an abattoir in Ohio

		Normal health outcome			Poor health outcome		
Health outcome^1^	Total animals	n	%	95% CI	n	%	95% CI
Arthritis	420	412	98.1	(95.7, 99.4)	8	1.90	(0.65, 4.29)
Broken ribs or tail	419	395	94.3	(90.3, 98.3)	24	5.73	(1.70, 9.75)
Dehydration	420	132	31.4	(19.9, 43.0)	288	68.6	(57.0, 80.1)
Depression	420	348	82.9	(78.1, 87.6)	72	17.1	(12.4, 21.9)
Diarrhoea	420	356	84.8	(80.0, 89.6)	64	15.2	(10.5, 20.0)
Thin body condition	417	251	60.2	(46.6, 73.9)	166	39.8	(26.1, 53.5)
Failed transfer of passive immunity	419	321	76.6	(71.7, 81.5)	98	23.4	(18.5, 28.3)
Fever	419	380	90.7	(85.5, 95.9)	39	9.31	(4.10, 14.5)
Hypoglycaemia	349	91	26.1	(18.7, 33.4)	258	73.9	(66.6, 81.3)
Navel inflammation	420	312	74.3	(70.4, 78.2)	108	25.7	(21.8, 29.6)
Respiratory disease	420	376	89.5	(86.3, 92.7)	44	10.5	(7.25, 13.7)

1 Poor health outcomes were considered for arthritis (joint score ≥ 2), broken ribs or tail (score = 1), dehydration (score ≥ 1), depression (score ≥ 1), diarrhoea (faecal score = 1), thin body condition (body condition score = 0), failed transfer of passive immunity (serum total protein < 5.1 g dL^–1^), fever (rectal temperature ≥ 39.4°C), hypoglycaemia (blood glucose < 4.95 mmol L^–1^), navel inflammation (score ≥ 2), respiratory disease (eyes, ears, or nose scores ≥ 2).

### Risk factors for poor health outcomes on arrival

Within our study population, nearly half of the calves (48.1%; 202/420) were female; Table 5 shows the number and percentage of female and male calves with poor health outcomes on arrival at the abattoir. Calf sex was a risk factor for hypoglycaemia (*P* < 0.0001); male calves were 3.1 times more likely to have blood glucose concentrations below the 4.95 mmol L^–1^ threshold than female calves (95% CI: 1.82 to 5.15). Sex also tended (*P* = 0.08) to be a risk factor for navel inflammation; male calves were 1.5 times more likely to have navel inflammation compared to female calves (95% CI: 0.96 to 2.38).

**Table 5.** Number and percentage of female and male calves with poor health outcomes from a sample of 420 bob veal calves on arrival at an abattoir in Ohio

	Female			Male		
Health outcomes^1^	Total animals	n	%	Total animals	n	%
Arthritis	202	33	16.4	218	40	18.4
Broken ribs or tail	202	10	5.0	218	15	6.9
Dehydration	202	134	66.7	218	154	70.6
Depression	202	34	16.9	218	38	17.4
Diarrhoea	202	31	15.4	218	33	15.1
Thin body condition	200	83	41.3	217	85	39.0
Failed transfer of passive immunity	201	43	21.4	218	55	25.2
Fever	201	18	9.0	218	21	9.6
Hypoglycaemia	162	102	63.0	187	156	83.4
Navel inflammation	202	43	21.4	218	65	29.8
Respiratory disease	202	18	9.0	218	26	11.6

1 Poor health outcomes were considered for arthritis (joint score ≥ 2), broken ribs or tail (score = 1), dehydration (score ≥ 1), depression (score ≥ 1), diarrhoea (faecal score = 1), thin body condition (body condition score = 0), failed transfer of passive immunity (serum total protein < 5.1 g dL^–1^), fever (rectal temperature ≥ 39.4°C), hypoglycaemia (blood glucose < 4.95 mmol L^–1^), navel inflammation (score ≥ 2), respiratory disease (eyes, ears, or nose scores ≥ 2).

Hydration status was significantly influenced by breed (*P* = 0.008). Jersey (OR: 0.42, 95% CI: 0.19 to 0.94) and crossbreed (OR: 0.27, 95% CI: 0.11 to 0.64) calves were less likely to be dehydrated than Holstein-Friesian calves. Breed was also a significant risk factor for thin body condition (*P* = 0.04). Jersey (OR: 0.35, 95% CI: 0.15 to 0.82) and crossbreed (OR: 0.42, 95% CI: 0.16 to 1.11) calves were less likely to have a Body Condition Score of 0 compared to Holstein-Friesian calves. Dehydration (using a skin tent test) and Body Condition Score were moderately correlated (*r* = 0.24; *P* < 0.0001).

Calves were sourced from three buying stations; Buying Station 1 was in New York (694 km from abattoir); Buying Station 2 was in Pennsylvania (410 km); and Buying Station 3 was in Ohio (11 km; Table 1). Source tended to be a risk factor for FTPI (*P* = 0.06), with calves arriving from Buying Station 2 having lower odds (OR: 0.52, 95 % CI: 0.31 to 0.89) of FTPI compared to calves sourced from Buying Station 3.

## Discussion

The objectives of this study were to: (i) assess the condition of surplus dairy calves destined for bob veal on arrival at an abattoir in Ohio; and (ii) determine risk factors for poor health outcomes. The most common health concerns on arrival at the abattoir were: hypoglycaemia, dehydration, thin body condition, and navel inflammation. Calf sex (male) was a risk factor for hypoglycaemia, and Holstein-Friesian calf breed was a risk factor for dehydration and thin body condition.

The majority of calves were considered dehydrated using a skin tent test on arrival at the abattoir. Our estimate of dehydration (68.6%) exceeds previous estimates in young calves on arrival at the abattoir (Roadknight *et al.* 2021a: 11% dehydration) and calf-rearing facilities (Pempek *et al.* 2017: 35% dehydration; Scott *et al.* 2019: 32% dehydration). It is important to interpret these differences with care, as each studies’ evaluation of dehydration varied (e.g. Roadknight *et al.* 2021a: urea concentration > 7.7 mmol L^–1^; Pempek *et al.* 2017: skin tent > 4 s; Scott *et al.* 2019: ≥ 2 s).

Typically, calves are not provided feed and water during marketing or transport (United States: Pempek *et al.* 2017; reviewed by Creutzinger *et al.* 2021; Australia: Roadknight *et al.* 2021b), and the level of care (e.g. colostrum management, feed, water) provided on the source dairy farm prior to sale is not well-documented. Indeed, there is some evidence that males receive poorer care after birth compared to females that remain on the dairy farm as replacements for the milking herd (Shivley *et al.* 2016; Renaud *et al.* 2017). Time off feed and water for young calves during marketing and transport is generally unknown and could be extensive, particularly if calves are sold multiple times before arriving at their destination. In addition to these factors, calves may experience disease and extreme thermal conditions, which could also influence the high prevalence of dehydration among surplus calves (Pempek *et al.* 2017). It should also be noted that calves likely feel thirsty prior to the onset of clinical signs of dehydration; this, too, should be considered to promote calf welfare prior to arrival at the abattoir or calf-rearing facilities.

One possible difference between the high prevalence of dehydration in our study compared to others might relate to differences in transport regulations between countries. For instance, Australian calves must be at least five days old before transport, with a maximum transport time of 12 h before animals must be offloaded to rest (Australia Animal Health 2012); whereas the United States government does not have regulations on calf age at transport, and the maximum transport time before animals must be offloaded to rest is 28 h (United States Government 2011). Our findings support previous results (Pempek *et al.* 2017; Scott *et al.* 2019) that calves are not receiving sufficient fluids before or during transport between the dairy farm of birth and their destination (Pempek *et al.* 2017). We strongly recommend that calves are provided milk or an oral electrolyte solution on the dairy farm prior to sale, as well as during marketing (e.g. live action, buying station) to reduce calves’ risk of dehydration, hypoglycaemia and, ultimately, compromised welfare.

There is a possibility that the high prevalence of dehydration in our study led to inflated STP values and, ultimately, a suppressed prevalence of FTPI (Tyler *et al.* 1999). Nonetheless, STP has been shown to be a reliable estimate of TPI and can be used despite a high prevalence of mild dehydration (Renaud *et al.* 2018b). Comparatively, the prevalence of FTPI (23.4%) in our study was similar to a recent Canadian study (24%; Renaud *et al.* 2020) considering young surplus dairy calves, but higher than previous studies (Windeyer *et al.* 2014: 11% FTPI) and recent national estimates among heifer calves in the US (Shivley *et al.* 2018: 12.1%). It is important to note that there is no current mechanism in place to record or report calf-level care practices, such as colostrum management, navel antisepsis, etc, prior to calves entering different sectors of the surplus calf market (Creutzinger *et al.* 2021). It may be possible that if a dairy producer knows or assumes their surplus calves are destined for bob veal, then their calves may receive a lower standard of care because of the young age at which bob veal calves are harvested (< 3 weeks of age), compared to calves entering special-fed veal and dairy beef sectors of the surplus calf industry. Future social science research is necessary to understand producer attitudes towards different sectors of the calf industry, as well as strategies to improve colostrum management.

Male calves had higher odds of arriving at the abattoir with hypoglycaemia and navel inflammation, indicating differences in calf-care practices based on sex. A lower standard of care for male calves after birth has been noted previously (United States: Shivley *et al.* 2019; Canada: Scott *et al.* 2019), and social science efforts are being made to better understand why care discrepancies exist between male and female calves (Wilson *et al.* 2021). Surplus calf markets rely heavily on calves receiving a high-level of care on the dairy farm of birth, regardless of whether they are destined for the abattoir or calf-rearing facilities, to mitigate potential welfare concerns. However, surplus calf care requires high-value resources (e.g. time, money) with often little to no financial pay-off for dairy producers in the current economic climate (Creutzinger *et al.* 2021). Differences in calf condition based on sex in our study could also be due to differences in marketing practices between male and female calves; records on calf source from the abattoir indicated only the last known point of sale for calves in our study. It is possible that male calves were sold multiple times, leading to a longer time off feed and exposure to different environments or pathogens, influencing the prevalence of calves with hypoglycaemia and navel inflammation, respectively. Future efforts in the United States are necessary to better track inter- and intra-state movements of surplus calves to better understand this finding and calf movements.

Breed was a significant risk factor for dehydration and thin body condition. Jerseys and crossbreed calves were less likely to be dehydrated than Holstein-Friesian calves in our study. It is possible that skin elasticity is different between breeds, causing Holstein-Friesian calves to appear more dehydrated when using a skin tent test. Future studies should evaluate dehydration in young calves using different methodologies (e.g. blood biomarkers, clinical evaluation) across breeds to determine if breed plays a role in skin elasticity and dehydration estimates. It is also possible that the difference in dehydration between breeds is associated with on-farm management practices. Breed was also a risk factor for thin body condition in our study, which may be associated with hydration status, as they were moderately correlated. Since Jersey and crossbreed calves were less likely to be dehydrated, they may have experienced less shrink during transport and ultimately reduced the prevalence of thin body condition. It should be noted that body condition assessment in very young calves (e.g. < 1 week of age) should be interpreted with care, as there is limited time for young calves to either acquire or lose muscle and fat coverage, even if they experience different levels of care prior to arrival at the abattoir.

Many factors can influence calf condition prior to arrival at the abattoir, and a larger national study is necessary to benchmark calf condition on arrival at abattoirs across the US. The bob veal calf sector, as well as formula-fed veal and dairy beef sectors, would benefit from programmes similar to the National Beef Quality Audit (NBQA); the NBQA evaluates a variety of production parameters, benchmarking the beef industry’s progress at five-year intervals (BQA 2022). Opportunity exists for audits of surplus dairy calves that are national or international in scale to provide similar data and track progress across the bob veal, formula-fed veal, and dairy beef industries. Information generated through a more consolidated effort could be used to inform future research and calf care standards. Data collected could also help extension efforts communicate ways to reduce the prevalence of poor health outcomes, such as colostrum management to reduce FTPI or proper handling to decrease broken ribs and tails.

A possible limitation of this study was that calves experiencing very poor welfare may not have been included in the study, similar to Roadknight and colleagues (2021a). Since sampling occurred at the abattoir in the holding pens prior to harvest, only calves that were fit enough to survive transportation and walk unassisted off the trailer into the holding pens were included in our study population. Nonetheless, we were able to quantify the number of calves that were dead or euthanased on arrival at the abattoir; 1.4%. This is considerably higher than recent mortality estimates among bob veal calves in New Zealand (0.04%; New Zealand Government 2020). Our study’s mortality estimate may have been inflated from a smaller sample size, but it may also reflect differences in national legislation considering transport of bob veal calves (for a review, see Roadknight *et al.* 2021b). Background information on calves beyond their last known point of sale was not recorded by the abattoir and could also be considered a limitation of the current study.

### Animal welfare implications

Few studies have investigated morbidity and associated risk factors in surplus dairy calves prior to slaughter. To our knowledge, our study is the first to assess the condition of bob veal calves on arrival at an abattoir in the US. Similar to the few studies that have been published on surplus dairy calves in other industries (e.g. formula-fed veal) and countries (e.g. Australia, New Zealand), the majority of calves in our study were experiencing at least one poor health outcome on arrival at the abattoir. Although our study focused on the calves’ physical state, it is likely that pre-slaughter calf management practices (e.g. fasting, transport, etc), also largely influence affective states, including hunger, thirst, among other states. More research is encouraged to understand the affective state of calves at different stages in the production chain prior to slaughter. Understanding surplus calf welfare and addressing the underlying risk factors associated with poor welfare is necessary to promote calf welfare in each stage of the production chain: at the dairy farm of birth, during marketing through live auction or assembly at buying stations, during transport, and during lairage at the abattoir.

## Conclusion

The majority of surplus calves arriving at an abattoir in Ohio had at least one poor health outcome, with a moderate to high prevalence of hypoglycaemia, dehydration, thin body condition, navel inflammation, and FTPI. Calf sex was a risk factor for hypoglycaemia; breed was a risk factor for dehydration and thin body condition; and source was a risk factor for FTPI. We conclude that there are opportunities to improve calf condition prior to arrival at the abattoir, on the dairy farm of birth (e.g. colostrum management, navel care) and during marketing at live auction or collection points (e.g. by providing access to milk or oral electrolyte solutions). Future research should investigate evidence-based strategies to promote bob veal calf welfare throughout the surplus calf production chain, from birth on through to harvest.
